# Induction of cell cycle arrest and apoptosis in caspase-3 deficient MCF-7 cells by *Dillenia suffruticosa* root extract via multiple signalling pathways

**DOI:** 10.1186/1472-6882-14-197

**Published:** 2014-06-19

**Authors:** Jhi Biau Foo, Latifah Saiful Yazan, Yin Sim Tor, Nurdin Armania, Norsharina Ismail, Mustapha Umar Imam, Swee Keong Yeap, Yoke Kqueen Cheah, Rasedee Abdullah, Maznah Ismail

**Affiliations:** 1 Laboratory of Molecular Biomedicine, Institute of Bioscience, Universiti Putra Malaysia, 43400 UPM Serdang, Selangor, Malaysia; 2 Department of Biomedical Science, Faculty of Medicine and Health Sciences, Universiti Putra Malaysia, 43400 UPM Serdang, Selangor, Malaysia; 3 Laboratory of Vaccines & Immunotherapeutics, Institute of Bioscience, Universiti Putra Malaysia, 43400 UPM Serdang, Selangor, Malaysia; 4 Department of Pathology and Microbiology, Faculty of Veterinary Medicine, Universiti Putra Malaysia, 43400 Serdang, Malaysia

**Keywords:** *Dillenia suffruticosa*, Dichloromethane extract, Cell cycle, Apoptosis, Oxidative stress

## Abstract

**Background:**

*Dillenia suffruticosa* root dichloromethane extract (DCM-DS) has been reported to exhibit strong cytotoxicity towards breast cancer cells. The present study was designed to investigate the cell cycle profile, mode of cell death and signalling pathways of DCM-DS-treated human caspase-3 deficient MCF-7 breast cancer cells.

**Methods:**

*Dillenia suffruticosa* root was extracted by sequential solvent extraction. The anti-proliferative activity of DCM-DS was determined by using MTT assay. The mode of cell death was evaluated by using inverted light microscope and Annexin-V/PI-flow cytometry analysis. Cell cycle analysis and measurement of intracellular reactive oxygen species (ROS) were performed by using flow cytometry. MCF-7 cells were co-treated with antioxidants α-tocopherol and ascorbic acid to evaluate whether the cell death was mainly due to oxidative stress. GeXP-based multiplex system was employed to investigate the expression of apoptotic, growth and survival genes in MCF-7 cells. Western blot analysis was performed to confirm the expression of the genes.

**Results:**

DCM-DS was cytotoxic to the MCF-7 cells in a time-and dose-dependent manner. The IC_50_ values of DCM-DS at 24, 48 and 72 hours were 20.3 ± 2.8, 17.8 ± 1.5 and 15.5 ± 0.5 μg/mL, respectively. Cell cycle analysis revealed that DCM-DS induced G_0_/G_1_ and G_2_/M phase cell cycle arrest in MCF-7 cells at low concentration (12.5 and 25 μg/mL) and high concentration (50 μg/mL), respectively. Although Annexin-V/PI-flow cytometry analysis has confirmed that DCM-DS induced apoptosis in MCF-7 cells, the distinct characteristics of apoptosis such as membrane blebbing, chromatin condensation, nuclear fragmentation and formation of apoptotic bodies were not observed under microscope. DCM-DS induced formation of ROS in MCF-7 cells. Nevertheless, co-treatment with antioxidants did not attenuate the cell death at low concentration of DCM-DS. The pro-apoptotic gene *JNK* was up-regulated whereby anti-apoptotic genes *AKT1* and *ERK1/2* were down-regulated in a dose-dependent manner. Western blot analysis has confirmed that DCM-DS significantly up-regulated the expression of pro-apoptotic JNK1, pJNK and down-regulated anti-apoptotic AKT1, ERK1 in MCF-7 cells.

**Conclusion:**

DCM-DS induced cell cycle arrest and apoptosis in MCF-7 cells via multiple signalling pathways. It shows the potential of DCM-DS to be developed to target the cancer cells with mutant caspase-3.

## Background

Cancer is a polygenic and multifactorial disease. Cancer cells often acquire multiple gene mutations that allow the cells to escape the cell cycle regulation and programmed cell death [1]. The current understanding of the molecular cancer biology has revealed that cancers are subdivided into different pathways. In contrast to the traditional single molecule-single target approach, being able to target two or more pathways at once or a few players in the same pathway would be a more effective therapy for the treatment of cancer. This strategy is in parallel to the new guideline approved by US Food and Drug Administration (FDA) in 2011 which outlines a path towards developing combination therapy [1,2]. Thus, evidence-based herbal medicines might be one of the starts for these approaches. The mixture of active compounds in the herbal medicines may have synergistic effect on cancer such as targeting on several pathways, reducing adverse side effects, and altering drug metabolism and excretion [3,4]. The formulations of herbal medicine can be further explored which may lead to novel insights in pharmacological management of cancer and other diseases.

Mitogen-activated protein kinases (MAPKs) are a family of serine/threonine protein kinases involved in the early apoptosis that regulate important cellular processes such as cell survival and adaptation by phosphorylating numerous cytoplasmic and nuclear targets [5-8]. There are at least four distinct subgroups of MAPKs, of which the P38 mitogen-activated protein kinases (p38 MAPKs), c-Jun N-terminal kinases (JNKs), extracellular signal-regulated kinases (ERKs) and protein kinase B (PKB or AKTs) have been extensively described. Generally, p38 MAPKs and JNKs are involved in the induction of apoptosis, whereas ERKs and AKTs have been implicated as a factor involved in cell survival [9-13].

The transcription factor nuclear factor-kappa B (NF-κB) has been correlated to oncogenesis by upregulating the expression of survival factors of cancer cells, thereby inhibiting apoptosis. NF-κB is composed of hetero- or homodimers of the NF-κB/Rel family of proteins. NF-κB complexes are sequestered in the cytoplasm in an inactive form by their interaction with inhibitor of kB (IkB) proteins [14]. NF-κB activity is induced in response to a variety of stimuli including inflammatory cytokines, cellular stress, reactive oxygen species (ROS) or anticancer agents. The activation of IkB kinase (IKK) complexes phosphorylates IkB proteins, triggering their degradation via the proteasome. NF-κB complexes are then translocated into the nucleus, bind to specific DNA-binding sites and regulate the transcription of target genes [15] including various anti-apoptotic proteins, but also several pro-apoptotic molecules, consistent with reports that NF-κB can promote apoptosis under certain circumstances [16,17].

The search for signature genes of different cancers has shown that most cancers are due to dysregulation of multiple genes and multiple cell signalling pathways; thus, drugs that are multi-targeted (once called “dirty drugs”) are needed. Compounds from natural sources have an advantage in that they are usually multi-targeted [12]. *Dillenia suffruticosa* (Griffith ex Hook. F. and Thomson) Martelli (Family: Dilleniaceae), commonly known as “*Simpoh air*”, is found abundantly in the secondary forest and swampy ground of Malaysia. This plant is traditionally used as alternative herbal medicine for the treatment of cancerous growth including breast cancer [18]. Nevertheless, there is no pharmacological study on the anti-breast cancer properties of the root extract in the literature. In addition, the plant has also been reported to exhibit antimicrobial [19] and antiviral properties [20]. Previous studies conducted in our lab revealed that the root hot water extract of *D. suffruticosa* exhibited anti-cervical and colon cancer properties in rodents (Patent ID: 20120003490) [21]. In addition, root dichloromethane total extract of *D. suffruticosa* (DCM-DS) from sequential solvent extraction exhibited strong cytotoxicity towards human MCF-7 breast cancer cells [22]. Therefore, DCM-DS has a great potential to be developed as evidence-based complementary and alternative medicine for the treatment of breast cancer. Nevertheless, the underlying mechanisms of DCM-DS-induced cytotoxicity in caspase-3 deficient MCF-7 breast cancer cells remain to be elucidated. This study investigated the cell cycle profile, mode of cell death and signalling pathways of DCM-DS-treated human caspase-3 deficient MCF-7 breast cancer cells.

## Methods

### Plant material

Fine powder of *Dillenia suffruticosa* was supplied by Primer Herber Sdn. Bhd., Malaysia. The plant’s authentication was performed with the parts of the plants (flower, leaves, stems and roots) at the Biodiversity Unit, Institute of Bioscience, Universiti Putra Malaysia, Malaysia (voucher specimen number SK1937/11).

### Preparation of plant extract

DCM-DS from sequential solvent extraction exhibited strong cytotoxicity towards human MCF-7 breast cancer cells [22]. Therefore, DCM-DS was employed for the current study with modification on the extraction method (Patent ID: 20120003490). Briefly, 100 g of the powdered root was macerated with 500 μL of hexane (1:5, w/v) (Friedemann Schmidt, Francfort, Germany) for 2 days at room temperature with occasional shaking at 200 rpm (IKA KS 260 basic, IKA, Staufen, Germany). The mixture was then centrifuged at 2000 × *g* for 5 min. The supernatant was filtered through Whatman filter paper No. 1. The residue was re-extracted until the colour disappeared, dried in the oven (40°C for 24 hours) and further macerated with dichloromethane (DCM) (Friedemann Schmidt, Francfort, Germany). The combined DCM total extracts were pooled and DCM was removed under reduced pressure (Rotavapor R210, Buchi, Flawil, Switzerland). The percentage of yield for DCM-DS was calculated as: (weight of extract/weight of powdered root) × 100%.

### Cell culture

The human MCF-7 breast cancer and non-tumourigenic MCF10A cell lines were purchased from the American Type Culture Collection (ATCC, Manassas, VA, USA). MCF-7 cells were grown in phenol-red-free RPMI 1640 with L-glutamine (Nacalai Tesque, Kyoto, Japan), supplemented with 10% foetal bovine serum (FBS) (PAA, Pasching, Austria) and 1% penicillin–streptomycin (PAA, Pasching, Austria). MCF-10A cells were cultured in DMEM/F12 (Sigma-Aldrich, St. Louis, MO, USA) supplemented with 10% FBS (PAA, Pasching, Austria), 20 ng/mL epidermal growth factor (Sigma-Aldrich, St. Louis, MO, USA), 0.5 mg/mL hydrocortisone (Sigma-Aldrich, St. Louis, MO, USA), 100 ng/mL cholera toxin (Sigma-Aldrich, St. Louis, MO, USA), and 10 μg/mL insulin (Sigma-Aldrich, St. Louis, MO, USA). The cells used for each experiment were of less than 20 passage number.

### Determination of cell viability

The stock concentration (30 mg/mL) of DCM-DS total extract was prepared in dimethyl sulfoxide (DMSO) (Friedemann Schmidt, Francfort, Germany). MCF-7 and MCF-10A cells were trypsinized (trypsin-EDTA (1×), PAA, Pasching, Austria) and seeded in 96-well flat-bottomed plates with 5000 cells per well in 100 μL of complete growth culture media, followed by incubation at 37°C (5% CO_2_ and 95% air) for 24 hours to allow cell attachment. The cells were then treated with either Tamoxifen (Sigma-Aldrich, St. Louis, MO, USA) or DCM-DS for 24, 48 and 72 hours. Control cells treated with 0.3% DMSO alone were also included. DMSO at 0.3% was not toxic to the MCF-7 cells in the present study (unpublished data). Following incubation, 20 μL of MTT (3-(4,5-dimethylthiazol-2-yl)-2,5-diphenyltetrazolium bromide, PhytoTechnology Laboratories, Kansas, USA) (5 mg/mL in PBS) was added into each well and the plate was incubated for 3 hours. The excess MTT was then aspirated and the formazan crystals formed were dissolved by 150 μL of DMSO. The absorbance, which was proportional to cell viability, was measured at 570 nm and a reference wavelength of 630 nm by using EL×800™ Absorbance Microplate Reader (BioTek Instruments Inc., Vermont, USA). Cell viability was calculated based on the following equation [23]:

$$\text{Cell}\;\text{viability}\;\left(\%\right)=\;\frac{OD_{570-630}\;\text{Treatment}}{OD_{570-630}\;\text{Control}}\times \;100$$

A graph of percentage of cell viability versus concentration of DCM-DS was plotted, and the concentration of DCM-DS which inhibited 50% of cellular growth as compared to the control (IC_50_ value) was determined [24].

### Morphological study

MCF-7 cells were seeded in 6-well plates at 1.3 × 10^5^ cells per well in 3 mL of complete growth medium, incubated for 24 hours and treated with DCM-DS (12.5-50 μg/mL). Control cells treated with 0.3% DMSO alone were also included. The morphological changes, characteristic of apoptosis or necrosis, were observed and the images were captured under an inverted light microscope (Olympus, PA, USA) at 0, 24, 48 and 72 hours. The same spot of cells was marked and captured.

### Cell cycle analysis

Cells were seeded in 6-well plates at 1.3 × 10^5^ cells in 3 mL of complete growth culture media, incubated for 24 hours and treated with DCM-DS (12.5-50 μg/mL). Following incubation, the floating cells were collected and adherent cells were harvested by trypsinisation to detach the cells and pelleted at 100 × *g* for 5 min. Cells were washed twice with PBS and resuspended in 70% ethanol at -20°C overnight. Prior to analysis, the cells were washed once with PBS, suspended in 425 μL of PBS, 25 μL of propidium iodide (1 mg/mL, Sigma-Aldrich, St. Louis, MO, USA) and 50 μL of RNaseA (1 mg/mL, Sigma-Aldrich, St. Louis, MO, USA), and incubated on ice for 20 min. The DNA content of 10000 cells was analysed by FACSCalibur flow cytometer (Becton Dickinson, CA, USA) and the population of cells in each cell-cycle phase was determined by using the ModFit LT software.

### Determination of apoptosis by Annexin V/PI staining

Experiment was carried out according to the manufacturer’s instructions (Annexin V-FITC kit, eBioscience, Vienna, Austria) with slight modification. Cells were seeded in 6-well plates at 1.3 × 10^5^ cells per well in 3 mL of complete growth culture media, incubated for 24 hours and treated with DCM-DS (12.5-50 μg/mL). Following incubation, the floating cells were collected and the adherent cells were trypsinized to detach the cells. Cells were counted and a volume of media containing 1 × 10^5^ cells (both viable and death cells) was centrifuged at 100 × *g* to obtain a pellet. Next, 195 μL of 1× assay buffer and 5 μL of Annexin V-fluorescein isothiocyanate (FITC) were added to the pellet. The samples were mixed by gentle tapping. Following 10 min incubation at room temperature in the dark, 300 μL of 1× assay buffer and 10 μL of PI (20 μg/mL) were added, and the samples were analysed immediately by FACSCalibur flow cytometer (Becton Dickinson, CA, USA). For each sample, 10000 events were collected. The results were analysed by using FlowJo software.

### Measurement of intracellular reactive oxygen species in DCM-DS-treated cells

Dichlorodihydrofluorescein diacetate (DCFH-DA, Sigma-Aldrich, St. Louis, MO, USA) was used to measure intracellular reactive oxygen species (ROS) in DCM-DS-treated cells. MCF-7 cells were seeded in 6-well plates at 1.3 × 10^5^ cells per well in 3 mL of complete growth culture media, incubated for 24 hours and pretreated with 10 μM DCFH-DA in FBS-free culture media for 1 hour. Next, the excess DCFH-DA was removed. The cells were then washed twice with PBS and further treated with DCM-DS in FBS-free culture media for 3 hours. Following incubation, the floating cells were collected and the adherent cells were trypsinized to detach the cells. The samples were then analysed immediately by FACSCalibur flow cytometer (Becton Dickinson, CA, USA). For each sample, 10000 events were collected. The results were analysed by using FlowJo software.

### Evaluation of antioxidants on DCM-DS-induced cell death in MCF-7 cells

MCF-7 cells were seeded in 96-well plates (5000 cells/well) and incubated at 37°C (5% CO_2_ and 95% air) for 24 hours. The cells were then treated with DCM-DS or co-treated with 50 μM α-tocopherol or vitamin C (Sigma-Aldrich, St. Louis, MO, USA) for 24 and 48 hours. At the end of the experiment, 20 μL MTT (5 mg/mL in PBS) was added into each well and the plate was incubated for 3 hours. The excess MTT was then aspirated and the purple formazan crystals were dissolved by 150 μL of DMSO. The absorbance was measured at 570 nm and a reference wavelength of 630 nm by using EL×800™ Absorbance Microplate Reader (BioTek Instruments Inc., Vermont, USA). Cell viability was calculated based on the following equation [23]:

$$\text{Percentage}\;\text{of}\;\text{cell}\;\text{viability}\;\left(\%\right)=\frac{OD_{570-630}\;\mathrm{Treatment}}{OD_{570-630}\;\mathrm{Control}}$$

A graph of percentage of cell viability versus concentration of DCM-DS was plotted, and the concentration of DCM-DS which inhibited 50% of cellular growth as compared to the control (IC_50_ value) was determined [24].

### RNA extraction

Cells were seeded in 6-well plates at 1.3 × 10^5^ cells per well in 3 mL of complete growth culture media. After treatment with DCM-DSE for 24 hours, the floating cells were collected and the adherent cells were trypsinised to detach the cells. The cells were centrifuged at 100 × *g* to obtain a pellet and washed twice with PBS. RNA was then isolated from the cells using the Real Genomics Total RNA Extraction Kit (RBCBioscience, Taipei, Taiwan). Briefly, the cells were mixed with 100 μL of lysis buffer, 400 μL of RB buffer and 4 μL of β-mercaptoethanol, and incubated for 5 min on ice. After incubation, 400 μL of 70% ethanol was added. Vigorous pipetting was performed to break any precipitate. The mixture was then transferred to RT column and centrifuged at 1000 × *g* for 2 min to bind RNA to the column. The RT column was transferred to a new collection tube, washed once with W1 Buffer and 2 times with Wash Buffer. Finally, the RT column was transferred to another new collection tube. RNAse-free water (50 μL) was added into the column matrix to dissolve the RNA. The RT column was centrifuged at 1000 × *g* for 1 min to elute purified RNA. The RNA concentration and quality were checked by a nano-photometer (Implen, Baxter Avenue, Britain).

### cDNA synthesis

RNA was reverse transcribed with multiplex universal reverse primers (Table 1) according to the GenomeLab™ GeXP Start Kit (Beckman Coulter Inc, CA, USA) from Beckman Coulter protocol with slight modification. Briefly, 50 ng of RNA (1 μL) from each sample was mixed with 1 μL of KAN^r^ RNA, 1 μL of reverse transcriptase, 2 μL of multiplex universal reverse primers, 4 μL of 5× reverse transcription buffer and 11 μL of RNAse-free water. The total volume for each sample was 20 μL. The reverse transcription was performed in a XP Thermal Cycler (Bioer Technology, Hangzhou, China) with the following mode: 48°C for 1 min; 42°C for 60 min; 95°C for 5 min and hold at 4°C.

**Table 1:** Genes used in GeXP multiplex analysis

**Name**	**Accession number**	**Left sequence w/Universals**	**Right sequence w/Universals**
*JNK*	NM_139046	AGGTGACACTATAGAATACAGAAGCTCCACCACCAAAGAT	GTACGACTCACTATAGGGAGCCATTGATCACTGCTGCAC
*18SRNA*^a^	M10098	AGGTGACACTATAGAATAGGAGTGGAGCCTGCGGCTTAA	GTACGACTCACTATAGGGATAGCATGCCAGAGTCTCGTT
*ERK1/2*	NM_002745	AGGTGACACTATAGAATAGGAGCAGTATTACGACCCGA	GTACGACTCACTATAGGGAGATGTCTGAGCACGTCCAGT
*AKT1*	NM_001014431	AGGTGACACTATAGAATAGAGGAGATGGACTTCCGGTC	GTACGACTCACTATAGGGAAGGATCTTCATGGCGTAGTAGC
*NF-κB*	NM_001077493	AGGTGACACTATAGAATAGCGGGCGTCTAAAATTCTG	GTACGACTCACTATAGGGATTCCACGATCACCAGGTAGG
*ACTB*^a^	NM_001101	AGGTGACACTATAGAATAGATCATTGCTCCTCCTGAGC	GTACGACTCACTATAGGGAAAAGCCATGCCAATCTCATC
*GAPDH*^a^	NM_002046	AGGTGACACTATAGAATAAAGGTGAAGGTCGGAGTCAA	GTACGACTCACTATAGGGAGATCTCGCTCCTGGAAGATG
*p38MAPK*	NM_001315	AGGTGACACTATAGAATATTCAGTCTTTGACTCAGATGCC	GTACGACTCACTATAGGGAGTCAGGCTTTTCCACTCATCT
*Kan(r)*^b^		AGGTGACACTATAGAATAATCATCAGCATTGCATTCGATTCCTGTTTG	GTACGACTCACTATAGGGAATTCCGACTCGTCCAACATC
*SOD1*	NM_000454	AGGTGACACTATAGAATATCATCAATTTCGAGCAGAAGG	GTACGACTCACTATAGGGATGCTTTTTCATGGACCACC
*SOD2*	NM_000636	AGGTGACACTATAGAATACATCAAACGTGACTTTGGTTC	GTACGACTCACTATAGGGACTCAGCATAACGATCGTGGTT
*CAT*	NM_001752	AGGTGACACTATAGAATAGAAGTGCGGAGATTCAACACT	GTACGACTCACTATAGGGAACACGGATGAACGCTAAGCT

^a^Gene used for normalization; ^b^Internal control gene.

### Polymerase chain reaction

Following reverse transcription, polymerase chain reaction (PCR) was performed to amplify the amount of cDNA according to the GenomeLab™ GeXP Start Kit (Beckman Coulter Inc, CA, USA) from Beckman Coulter protocol. cDNA from each sample (9.3 μL) was mixed with 2 μL of 200 nM forward universal primer mixture, 4 μL of 25 mM MgCl_2_, 4 μL of 5× PCR Master Mix buffer and 0.7 μL of Taq polymerase. The total volume for each sample was 20 μL. Amplification conditions consisted of 95°C for 10 min, followed by 35 cycles of 94°C for 30 sec, 55°C for 30 sec and 70°C for 1 min. The reactions were performed in a XP Thermal Cycler (Bioer Technology, Hangzhou, China).

### Gene multiplex data analysis

PCR product (l μL) was mixed with 38.5 μL of sample loading solution along with 0.5 μL of DNA Size Standard 400 (GenomeLab GeXP Start Kit, Beckman Coulter, Inc) and analysed on a GeXP genetic analysis system (S.Kraemer Boulevard, USA). The GeXPS system was used to separate PCR products based on size by capillary gel electrophoresis and to measure their dye signal strength in arbitrary units (A.U.) of optical fluorescence, defined as the fluorescent signal minus background.

### Fragment and gene expression signature analyses

The data were analysed using the Fragment Analysis module of the GeXP system software and imported into the analysis module of eXpress Profiler software. Fold change was normalised against beta actin*.*

### Western blot analysis

Bovine serum albumen (BSA), phosphatase inhibitor cocktails and Chemi-Lumi One L were purchased from Nacalai Tesque (Kyoto, Japan). Phenylmethanesulfonyl fluoride (PMSF) and protease inhibitor cocktails were purchased from Calbiochem (San Diego, CA, USA). Sodium dodecyl sulphate (SDS), Triton-X 100, Tris-base, glycine, acrylamide, bisacrylamide, ammonium persulfate (APS), tetramethylethylenediamine (TEMED), 10% Tween-20, Bradford Reagent, 2-mercaptoethanol, extra thick blotting paper and pre-stained protein marker were purchased from Bio-Rad (California, USA). Immobilon-FL polyvinylidene fluoride (PVDF) membrane with 0.45 μm pore size was purchased from Millipore (Bedford, MA, USA). Rabbit anti-AKT1 (ab32505), rabbit anti-ERK1 (ab32537), rabbit anti-JNK1 (ab10664), rabbit anti-p-JNK1 (ab47337), rabbit anti-NF-ҡB p65 (ab7970) and mouse anti-beta-actin (ab8226) primary antibodies were purchased from ABCAM (Cambridge, MA, USA). Horseradish peroxidase-conjugated goat anti-rabbit (ab6721) and goat anti-mouse (ab97240) secondary antibodies were purchased from ABCAM (Cambridge, MA, USA).

MCF-7 cells were seeded into 75 cm^2^ tissue culture flasks at 800,000 cells per flask with 15 mL of complete growth culture media and incubated for 24 hours. After treatment with DCM-DS for 24 and 48 hours, the floating cells were collected in a 50 mL centrifuge tube and washed twice with cold PBS. The adherent cells were also washed twice with cold PBS, lysed with 200 μL of cold lysis buffer (50 mM Tris–HCl pH 7.4, 150 mM NaCl, 0.1% SDS (w/v), 1% Triton-X 100 (v/v), 0.5% sodium deoxycolate (w/v)). PMSF (1 mM), protease inhibitor cocktails (10 μL/mL of lysis buffer) and phosphatase inhibitor cocktails (10 μL/mL of lysis buffer) were added to the cold lysis buffer prior to lysis of the cells. The flasks were then kept on ice for 5 min with occasionally swirling the flasks for uniform spreading of the lysis buffer. The lysate was gathered at one side using a cell scraper, collected with 1 mL pipette, pooled with the floating cells, transferred to a microcentrifuge tube and centrifuged at 14,000 × *g* for 10 min to pellet the cell debris. The clarified supernatant was then collected and stored at -80°C. The protein concentration quantification was performed by using Bradford Protein Assay. An equal amount of 10–20 μg of proteins was separated by 12% SDS-PAGE. After electrophoresis, the proteins were transferred to PVDF membrane by semi-dry transfer method, blocked with 3% BSA in 0.1% Tween-20 containing Tris-Buffer Saline (TBS-T) at room temperature (20–25°C) for 1 hour, reacted with anti-AKT1 (1:10,000), anti-ERK1 (1:5000), anti-JNK1 (1:10,000), anti-p-JNK1 (1:5000), anti-NF-ҡB p65 (1:5000) and anti-beta-actin (1:10,000) primary antibodies in TBS-T overnight at 4°C. After washing 3 times with TBS-T at room temperature, the primary antibodies were either reacted with horseradish peroxidase-conjugated goat anti-rabbit (1:40,000) or goat anti-mouse (1:40,000) secondary antibodies in TBS-T for 1 hour at room temperature. The protein visualisation was then performed by using Chemi-Lumi One L and ChemiDoc™ MP System (Bio-Rad, Hercules, CA, US) in a dark room.

### Statistical analysis

Statistical analysis was performed using the Statistical Package for Social Science (SPSS) version 21.0. Data were expressed as mean ± standard deviation (mean ± SD). Results were analysed by one-way analysis of variance (ANOVA), followed by Post Hoc Multiple Comparisons. A difference was considered to be significant at p < 0.05.

## Results

### DCM-DS was cytotoxic and inhibited growth of MCF-7 cells

**Figure 1:**
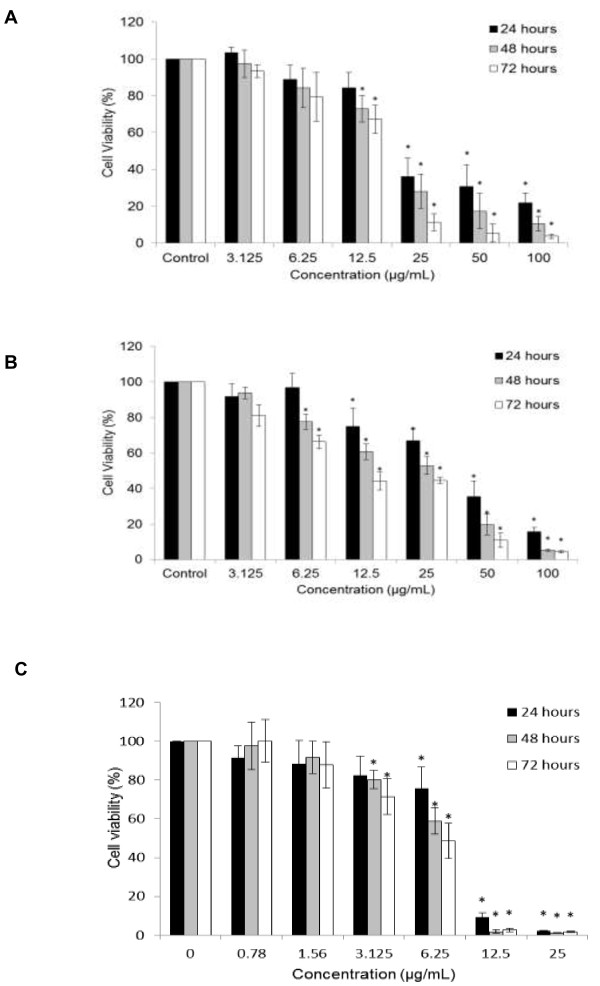
**Effect of DCM-DS on the viability of MCF-7 breast cancer and non-tumorigenic MCF10A cells as determined by MTT assay.** Cytotoxic effect of DCM-DS was evaluated on **(A)** MCF-7 and **(B)** MCF-10A cells. The extract was cytotoxic to the cells in a dose- and time-dependent manner. **(C)** Antiproliferative effect of Tamoxifen on MCF-7 cells . Each data point represents the mean of three independent experiments ± SD. *significantly different from the control (p < 0.05).

**Figure 2:**
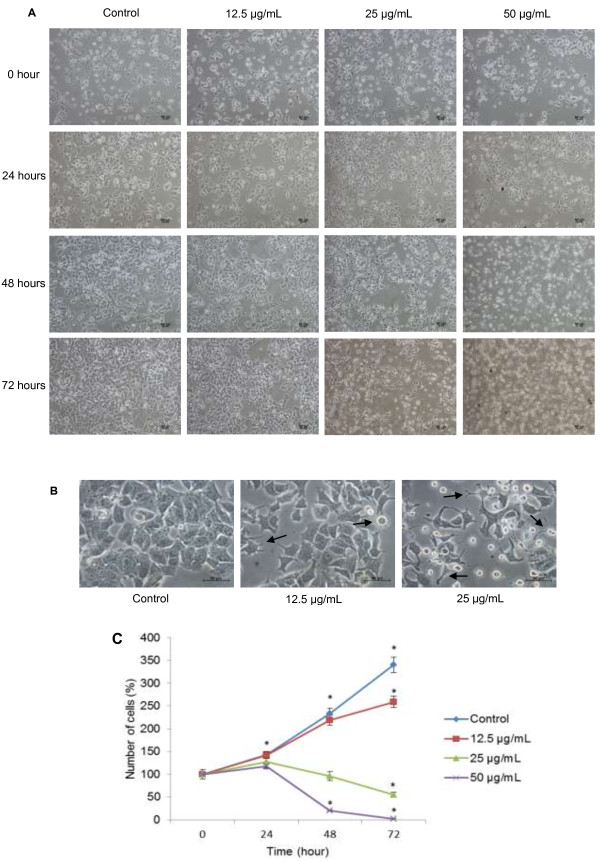
**DCM-DS inhibited the growth of MCF-7 cells. (A)** Morphological changes of MCF-7 cells after treatment with DCM-DS (100×) **(B)** Effect of DCM-DS on MCF-7 cells at 48 hours (400×). Cellular shrinkage was observed in DCM-DS-treated MCF-7 cells (arrows). **(C)** Changes in number of cell (percentage) treated with DCM-DS for 24, 48 and 72 hours. * is significantly different (p < 0.05).

DCM-DS was cytotoxic towards MCF-7 cells in a dose- and time-dependent manner (Figure 1A). Treatment with DCM-DS at higher concentrations (12.5-100 μg/mL) resulted in significant reduction (p < 0.05) in the cell viability. The IC_50_ values of DCM-DS towards MCF-7 cells at 24, 48 and 72 hours were 20.3 ± 2.8, 17.8 ± 1.5 and 15.5 ± 0.5 μg/mL, respectively. The IC_50_ value of Tamoxifen towards MCF-7 cells at 72 hours was 7.0 ± 1.0 μg/mL. In addition, DCM-DS was also cytotoxic towards MCF-10A cells in a dose- and time-dependent manner (Figure 1B). The IC_50_ values of DCM-DS towards MCF-10A cells at 24, 48 and 72 hours were 37.6 ± 4.3, 24.0 ± 2.2 and 10.3 ± 2.1 μg/mL, respectively.Morphological study revealed that DCM-DS induced growth inhibition and apoptosis in MCF-7 cells. As shown in Figure 2A and 2C, the number of cells in the control and the one treated with 12.5 μg/mL of DCM-DS increased from 0 to 72 hours. Nevertheless, the cells in the latter demonstrated cellular shrinkage at 48 hours (Figure 2B) and this phenomenon became more obvious at 72 hours. For the cells treated with DCM-DS at 25 μg/mL, increase in the cell population was noted until 24 hours. In the following hour, the growth was inhibited and the cells detached from the substratum (Figure 2A and 2B). Majority of the cells treated with DCM-DS at 50 μg/mL detached from the substratum at 48 and 72 hours (Figure 2A). Other characteristics of apoptosis such as membrane blebbing, chromatin condensation, nuclear fragmentation and formation of apoptotic bodies or necrosis were not observed (Figure 2B).

### DCM-DS induced G_0/_G_1_ and G_2_/M phase arrest in MCF-7 cells

**Figure 3:**
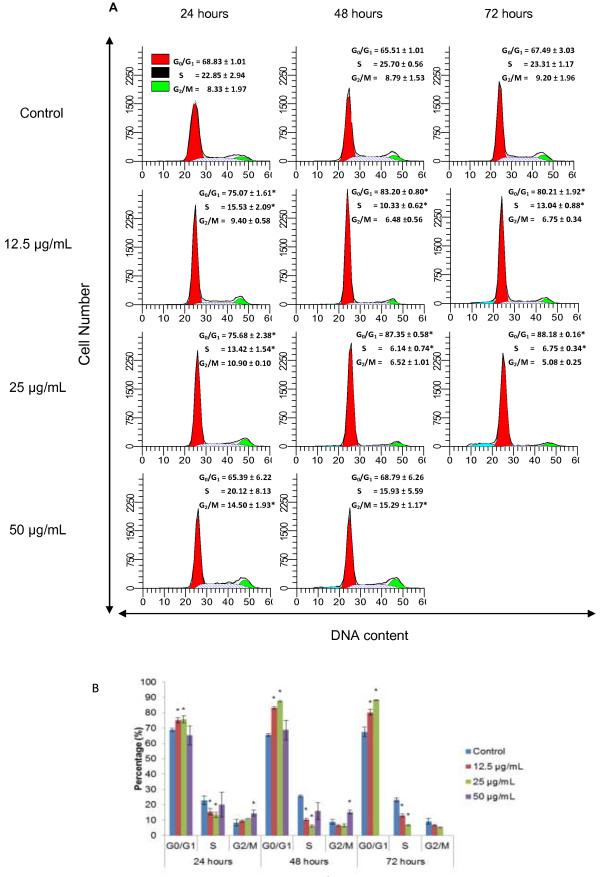
**Cell cycle profile of MCF-7 cells treated with DCM-DS (A and B).** The cell cycle profile of 50 μg/mL at 72 hours was not able to be determined due to the extensive formation of cell debris. Each data point represents the mean of three independent experiments ± SD. *significantly different from the control (p < 0.05).

Treatment with DCM-DS at 12.5 and 25 μg/mL significantly increased (p < 0.05) the population of cells at G_0_/G_1_ phase with a concomitant decrease (p < 0.05) in the S phase as compared to the control (Figure 3). A significant increase (p < 0.05) in the number of cells at G_2_/M phase was noted at 50 μg/mL of DCM-DS. The sub-G1 population (<10%) was observed at 72 hours of 12.5 and 25 μg/mL DCM-DS.

### DCM-DS induced apoptosis in MCF-7 cells

**Figure 4:**
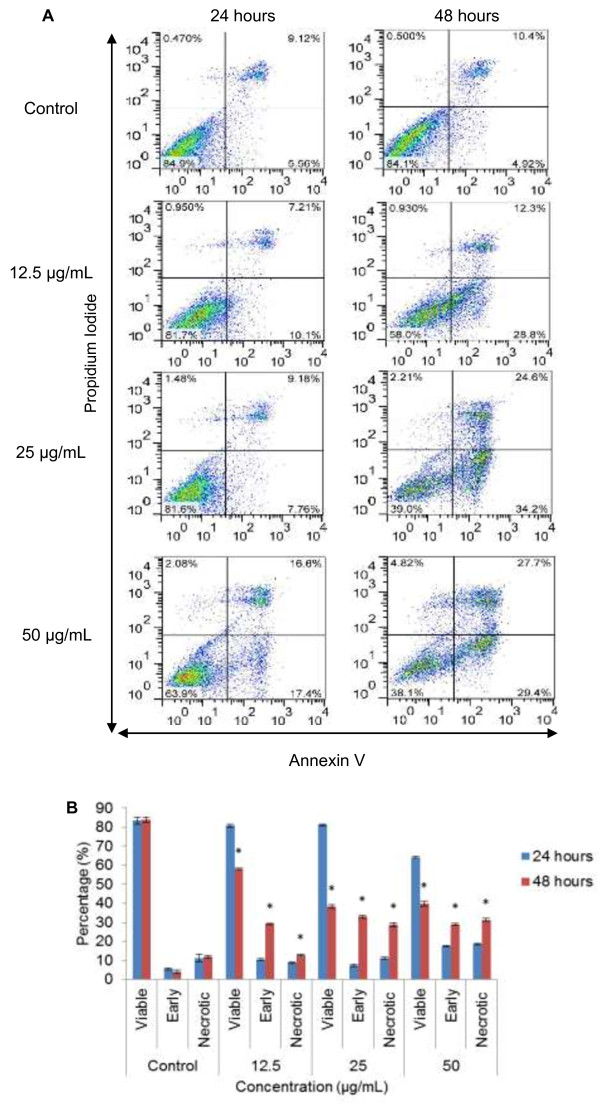
**The percentage of viable, apoptotic and necrotic/secondary necrotic cells of untreated and DCM-DS-treated MCF-7 cells for 24 and 48 hours as determined by flow cytometry. (A** and **B)** These figures are from representative experiments carried out at least three times. The percentage of viable cells was represented by the lower left quadrant (Annexin-V^-^/PI^-^); the percentage of early apoptotic and necrotic/secondary necrotic cells were represented by the lower right (Annexin-V^+^/PI^-^) and upper (PI^+^) quadrants, respectively. Each data point represents the mean of three independent experiments ± SD. *significantly different from the control (p < 0.05).

Treatment of MCF-7 cells with DCM-DS significantly increased (p < 0.05) the percentage of early apoptotic cells (Annexin-V^+^/PI^-^) as compared to the control (Figure 4A and 4B). The percentage of early apoptotic cells for 12.5, 25 and 50 μg/mL at 48 hours were 29.1, 32.9 and 28.8%, respectively, as compared to the control (4.0%) (Figure 4B). The percentage of dead cells or late apoptotic cells (PI^+^) for 12.5, 25 and 50 μg/mL at 48 hours were 12.8, 28.8 and 31.3%, respectively, as compared to the control (11.9%).

### DCM-DS induced formation of intracellular ROS in MCF-7 cells

**Figure 5:**
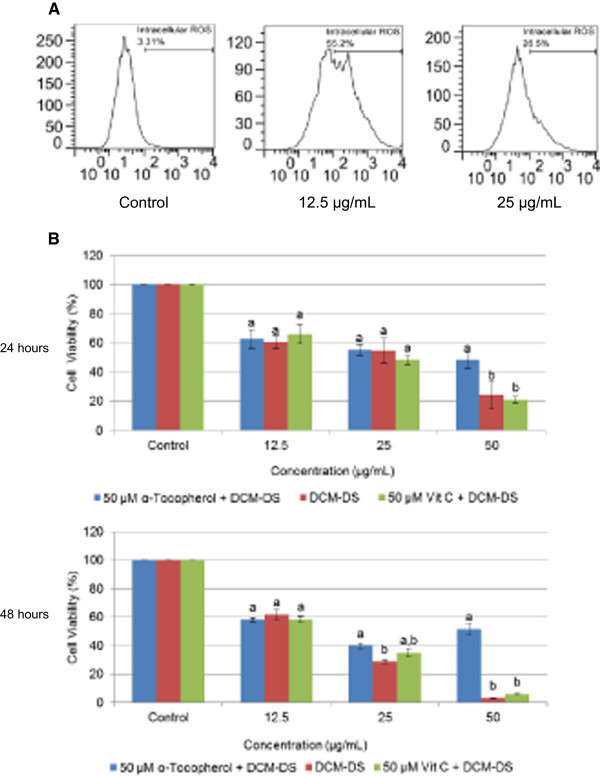
**Determination on the involvement of ROS in the cytotoxicity of DCM-DS in MCF-7 cells. (A)** Measurement of intracellular ROS in DCM-DS-treated MCF-7 cells by using DCFH-DA (Sigma-Aldrich). **(B)** Cell viability of MCF-7 cells co-treated with α-tocopherol or ascorbic acid for 24 and 48 hours. Each data point represents the mean of three independent experiments ± SD. a and b were significantly different among the concentration at the same time point (p < 0.05).

As shown in Figure 5A, the cells treated with DCM-DS at 12.5 and 25 μg/mL significantly increased (p < 0.05) the percentage of cells that expressed ROS to 55 and 26%, respectively, as compared to the 3% basal level.

### ROS partially induced cell death in MCF-7 cells

As shown in Figure 5B, the viability of the cells treated with DCM alone at 50 μg/mL for 24 and 48 hours were 24% and 3%, respectively. The co-treatment with 50 μM antioxidant α-tocopherol and DCM-DS significantly increased (p < 0.05) the viability of the cells to 48% and 50%, respectively. At 25 μg/mL of DCM-DS, co-treatment with α-tocopherol significantly increased the viability of the cells from 28% to 40% at 48 hours. In comparison, cells co-treated with 50 μM ascorbic acid did not increase or decrease in the viability of the cells at all the tested concentrations.

### DCM-DS regulated the apoptotic, growth and survival genes in MCF-7 cells

**Figure 6:**
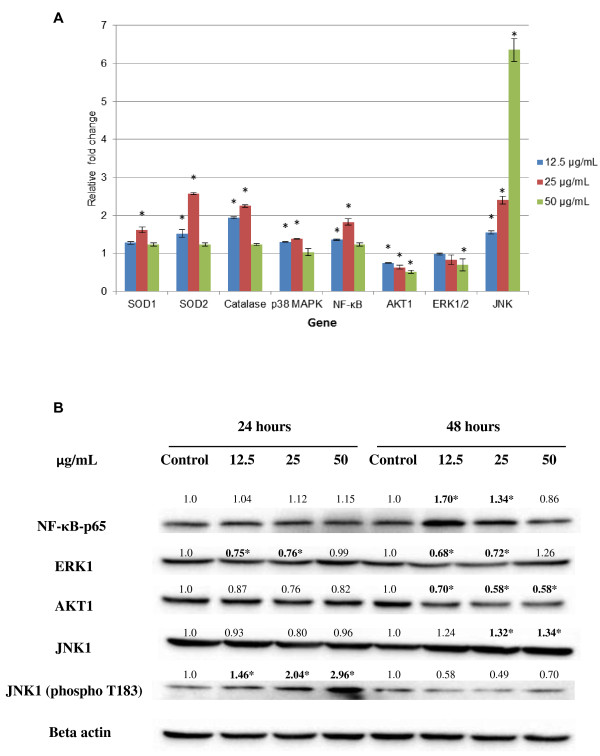
**Expression levels of genes and proteins in MCF-7 cells following treatment with DCM-DS. (A)** Relative fold change of gene expression in DCM-DS-treated MCF-7 cells at 24 hours as compared to the control. **(B)** Western blot analysis of the expression of NF-κB, JNK1, pJNK1, AKT1, ERK1and beta-actin from DCM-DS-treated MCF-7 cells. Fold change was normalised against beta-actin and compared to the control*.* Each data point represents the mean of three independent experiments ± SD. *significantly different from the control (p < 0.05).

Treatment of MCF-7 cells with DCM-DS significantly up-regulated (p < 0.05) the expression of *SOD1*, *SOD2*, *CAT*, *p38MAPK*, *JNK* and *NF-κB* in MCF-7 cells. On the other hand, the expression of *AKT1* and *ERK1/2* were significantly (p < 0.05) down-regulated in a dose-dependent manner (Figure 6A).

### DCM-DS up-regulated apoptotic protein and down-regulated anti-apoptotic proteins in MCF-7 cells

Treatment of MCF-7 cells with DCM-DS significantly up-regulated (p < 0.05) the expression of NF-κB, JNK1 and pJNK1. In addition, the expression of both AKT1 and ERK1 were down-regulated by the treatment (Figure 6B).

## Discussion

Breast cancer is the most frequent cancer among women. Approximately 1.67 million new breast cancer cases were reported in 2012, which accounts for 25% of all new diagnosed cancer [25]. The current treatment strategies for breast cancer patients such as surgery, chemotherapy and radiotherapy or the combination of radiotherapy and chemotherapy have successfully increased the five-year survival rate of the breast cancer patients. Nevertheless, the long-term survival remains poor due to cancer relapse and metastasis. *D. suffruticosa* has a great potential as an anticancer agent due to its traditional use for treatment of cancerous growth [18] and based on our previous findings [22]. In the present study, DCM-DS exhibited strong cytotoxic properties against caspase-3 deficient MCF-7 cells (Figure 1A). According to the National Cancer Institute guidelines, DCM-DS is a great candidate to be developed as an anticancer agent for breast cancer as the IC_50_ value was less than 20 μg/mL towards MCF-7 cells after treatment for 72 hours [26]. In addition, DCM-DS was also toxic to the non-tumourigenic MCF-10A cells (Figure 1B). Nevertheless, MCF-10A cells were more resistant to the treatment of DCM-DS as compared to the MCF-7 cells (the cell viability of MCF-10A treated with 25 μg/mL at 72 hours was 45% as compared to the 15% cell viability in MCF-7 cells).

The morphological analysis has revealed that DCM-DS-treated MCF-7 cells experienced cellular shrinkage, suggesting induction of apoptosis in the cells (Figure 2A). Inhibition of the cell growth at 12.5 and 25 μg/mL of DCM-DS between 24 to 48 hours (Figure 2A) was due to cell cycle arrest at G_0_/G_1_ phase as being confirmed by the flow cytometry analysis. Nevertheless, DCM-DS at 50 μg/mL arrested the cells at G_2_/M instead of G_0_/G_1_ (Figure 3A and 3B) suggesting that the dose of an agent may influence the effects on cell cycle.

The induction of apoptosis by DCM-DS in MCF-7 cells was ascertained by the Annexin-V-FITC/PI-flow cytometry analysis (Figure 4A and 4B). Apoptosis is characterized by distinct biochemical features in elimination of damaged cells or tumour cells without causing inflammation [27]. The activation of enzymatic and catabolic processes in apoptosis thereby facilitate cell morphological changes such as externalization of plasma membrane phosphatidylserine (PS), cellular shrinkage, membrane blebbing, chromatin condensation, nuclear fragmentation and formation of apoptotic bodies [28,29]. PS is a phospholipid component which has a strong binding affinity towards Annexin-V [30]. In a normal cell, PS is positioned on the inner surface of the cell membrane and therefore inaccessible to Annexin-V. At an early stage of apoptosis, PS is translocated to the outside of the cell membrane and bind with Annexin-V. PS translocation is an irreversible event. The apoptotic bodies formed are eventually engulfed by phagocytes such as neutrophils and macrophages in *in vivo*. Since the *in vitro* system is lack of phagocytes, the membrane of the apoptotic bodies will rupture (also known as secondary necrosis) and accessible to the PI dye. Therefore, secondary necrotic cells are stained by both Annexin-V and PI [30].

Even though DCM-DS was confirmed to induce apoptosis in MCF-7 cells, some other distinct morphological features of apoptosis such as membrane blebbing, chromatin condensation, nuclear fragmentation and formation of apoptotic bodies were not observed (Figure 2B). Indeed, the sub-G1 population was also undetectable even at the viability as low as < 10% in the treatment of 50 μg/mL DCM-DS at 48 hours (Figure 3). These observations could be due to the caspase-3 deficiency in the MCF-7 cells that were used in the present study which has been confirmed by using gene expression analysis. The result showed that there was no detectable caspase-3 in MCF-7 cells at mRNA level (unpublished data). Caspase-3 is crucial for typical biochemical and morphological changes of cells undergoing apoptosis such as nuclear fragmentation. Several investigators have reported that MCF-7 cells originated from ATCC was caspase-3 deficiency and the typical characteristics of apoptosis were absent in this cell line upon treatment with apoptosis inducing agents [31-33]. Molecular study has uncovered the caspase-3 deficiency in this cell line is caused by a 47-base pair deletion within exon 3 of the *CASP-3* resulting in the skipping of this exon during pre-mRNA splicing and introduction of a premature stop codon at position 42 that completely abrogates translation of the *CASP-3* mRNA [33]. Nevertheless, sub-G1 population (<10%) was detectable following 72 hours exposure of DCM-DS at 12.5 and 25 μg/mL (Figure 3). It is then postulated that the caspase-3-deficient MCF-7 cells underwent aberrant nuclear and cytoplasmic destruction, leading to high molecular weight DNA fragmentation upon treatment with DCM-DS that could be mistaken for the typical apoptotic (low molecular) DNA fragmentation [31,34]. Since DCM-DS induced cell cycle arrest and apoptosis in the caspase-3-deficient-MCF-7 cells, it is suggested that the induction of apoptosis is via caspase-3-independent pathways.

Further investigation on molecular pathways involved in the induction of apoptosis by DCM-DS was performed by using GeXP-based multiplex system. Expression of *SOD1*, *SOD2* and *CAT* was up-regulated following treatment with DCM-DS suggesting the stimulation of oxidative stress via generation of ROS in MCF-7 cells [35] and this has been confirmed by evaluating the ROS level (Figure 5A). The up-regulation of several antioxidant defence genes such as *SOD* and *CAT* is to protect the cells from harmful ROS, keeping them in normoxidant state [36]. The production of ROS such as hydrogen peroxide (H_2_O_2_), superoxide radicals (O_2_^-^) and hydroxyl radicals (OH^-^) is a consequence of incomplete reduction of oxygen molecules in the mitochondrial electron transport chain during respiration which impaired the normal cellular functions [37]. *SOD1* and *SOD2* code for copper-zinc superoxide dismutase and manganese superoxide dismutase, respectively, which scavenge the O_2_^-^ into H_2_O_2_ and molecular oxygen (O_2_). H_2_O_2_ is then converted to water by either catalase (coded by *CAT*), glutathione peroxidase, or peroxiredoxins [38]. It has been well known for decades that H_2_O_2_ exerts dose-dependent effects on cellular activities, from growth stimulation at very low concentration to growth arrest, apoptosis, and eventually necrosis as the concentration increases. It seems that ROS play dual roles, either beneficial or deleterious, depending on the level in living cells [35,39]. To investigate whether the cell death in the present study was mainly due to ROS, the cells were co-treated with antioxidant α-tocopherol and ascorbic acid (Figure 5B). The results showed that α-tocopherol and ascorbic acid did not block the reduction of cell viability at 12.5 and 25 μg/mL of DCM-DS, suggesting that ROS did not involve in the event at these two concentrations. Nevertheless, at high concentration of DCM-DS (50 μg/mL), the co-treatment with α-tocopherol significantly blocked the cell death, suggesting that high concentration of DCM-DS induced the formation of ROS which played a significant role to induce cell death in MCF-7 cells.

The expression of *AKT1* gene and the protein (Figure 6) were down-regulated in the present study suggesting the involvement of Akt pathway in DCM-DS-induced apoptosis. Other studies have shown that apoptosis in MCF-7 cells was related to the inhibition of Akt signalling pathway after treatment with Wogonin or retinoic-acid [40,41]. Akt is a serine-threonine kinase that facilitates the control of balance between survival and apoptosis. The activation of *AKT1* has been reported to protect the cells from oxidative stress and orchestrate tumourigenesis in animal model [5-8]. JNK is a stress responsive kinase. The activation of JNK has been reported to induce apoptosis in various cancer cells including MCF-7 cells [10]. In this study, JNK gene was up-regulated more than 2 folds following treatment with DCM-DS which was then confirmed by the Western blot analysis, suggesting that the activation of JNK could be due to the bioactive compounds present in the DCM-DS, which in turn induced growth inhibition and apoptosis in MCF-7 cells [9-12].

The NF-κB was initially characterized as a central regulator in response to pathogens and viruses [42]. Although compelling experimental data have identified NF-κB as a tumour-promoting transcription factor, recent studies have unravelled the function of NF-κB as one of the key players of apoptosis [16,17]. It means that NF-κB could be pro-or-anti-apoptotic protein. It has been reported that up-regulation of AKT and ERK1/2, which in turn activates the NF-κB resulting in the metastasis of breast cancer cells to the bone [43]. In contrast, treatment with DCM-DS up-regulated the expression of NF-κB in MCF-7 cells despite of the down-regulation of AKT1 and ERK1/2. It has also been demonstrated that NF-κB pathway may function independently from Akt pathway [44]. Hence, it is postulated that although some crosstalks exist between Akt and NF-κB pathway in response to DCM-DS-induced oxidative stress in MCF-7 cells, but it appears that these two pathways can act independently. Nevertheless, the up-regulation of NF-κB in the current study remains to be elucidated.

**Figure 7:**
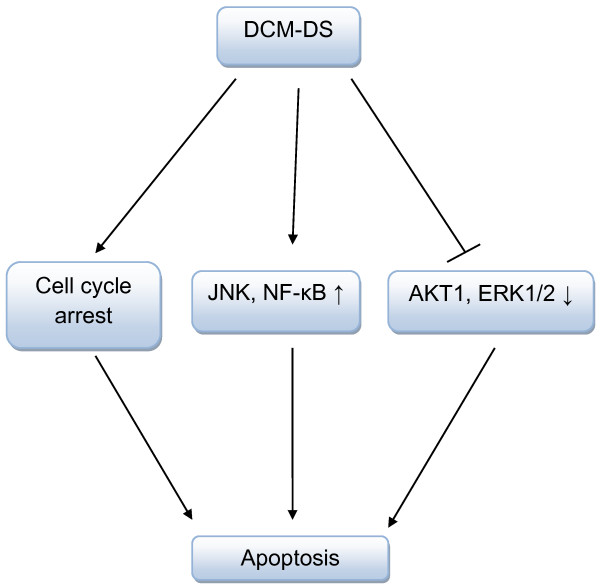
**Proposed schematic diagram of the mechanism by which DCM-DS induced apoptosis in caspase-3 deficient MCF-7 cells.** DCM-DS induced cell cycle arrest in MCF-7 cells. In addition, DCM-DS up-regulated NF-κB, JNK1 and down-regulated AKT1, ERK1, resulting in apoptosis in caspase-3 deficient MCF-7 cells.

The molecular mechanisms underlying the cytotoxicity of DCM-DS on MCF-7 cells are proposed in Figure 7. The shift of attention towards bioactive compounds and understanding of its mechanism of action are utmost essential to discover the potential of the extract in breast cancer intervention. Previous study reported that triterpenes appear to be the major compounds in DCM-DS that could be responsible to regulate the signalling pathways in the present study [22]. Several triterpenes such as betulinic acid and ursolic acid have been reported to induce apoptosis in cancer cells via regulation of MAPKs [13,45-48]. Nevertheless, a study will be carried out to isolate and characterise the bioactive compounds present in the DCM-DS that are responsible in the caspase-3 deficient MCF-7 cell death.

Breast cancer is a complex and heterogeneous disease that has been molecularly classified into 4 major subtypes: Luminal A, Luminal B, human epidermal growth receptor 2 (HER2) and basal-like breast cancers [49,50]. The caspase-3 deficient MCF-7 cells used in the present study fell to Luminal A subtype which accounts for approximately 40% of the total breast cancer cases [51]. Nevertheless, a future study should be conducted to evaluate the therapeutic effect of DCM-DS towards other breast cancer subtypes especially the basal-like breast cancer subtype which has the worst prognosis, metastasis to lung and brain, and high mortality rate (death within the first 3–5 years of diagnosis) as compared to other breast cancer subtypes [52,53].

## Conclusion

DCM-DS induced cell cycle arrest and apoptosis in caspase-3 deficient MCF-7 cells possibly via the up-regulation of NF-κB, JNK1 and down-regulation of AKT1 and ERK1. Therefore, DCM-DS has a great potential to be developed to target the cancer cells with mutant caspase-3.

## Competing interests

The authors declare that they have no competing interests.

## Authors’ contributions

JBF carried out the study and prepared the manuscript. JBF, YST and AN collected and interpreted the data. MUI and NI contributed to GeXP analysis. LSY, RA, YKC, SKY and MI contributed to the design and conception of the study and interpretation of data. LSY critically revised manuscript. All authors have read and approved the manuscript for publication.

## Pre-publication history

The pre-publication history for this paper can be accessed here:

http://www.biomedcentral.com/1472-6882/14/197/prepub
